# Factors Affecting Myocardial Infarction in Cervical Cancer Patients: A Population-Based Study

**DOI:** 10.4021/jocmr1591w

**Published:** 2013-10-12

**Authors:** Chen-Hsi Hsieh, Wen-Yen Chiou, Ching-Chih Lee, Moon-Sing Lee, Hon-Yi Lin, Yu-Chieh Su, Shih-Kai Hung

**Affiliations:** a Department of Radiation Oncology, Far Eastern Memorial Hospital, Taipei, Taiwan; b Department of Medicine, School of Medicine, National Yang-Ming University, Taipei, Taiwan; c Institute of Traditional Medicine, School of Medicine, National Yang-Ming University, Taipei, Taiwan; d Department of Radiation Oncology, Buddhist Dalin Tzu Chi General Hospital, Chiayi, Taiwan; e Department of Otolaryngology, Buddhist Dalin Tzu Chi General Hospital, Chiayi, Taiwan; f School of Medicine, Tzu Chi University, Hualian, Taiwan; g Department of Hematology Oncology, Buddhist Dalin Tzu Chi General Hospital, Chiayi, Taiwan; h These authors contributed equally to this work

**Keywords:** Cervical cancer, Myocardial infarction, Radiotherapy, Surgery

## Abstract

**Background:**

Radiotherapy (RT) or concurrent chemoradiation therapy has been suggested to increase the risk of coronary heart disease for cervical cancer patients, but the results of studies have been inconsistent. Therefore, we aimed to investigate the factors which influence the risk of developing myocardial infarction (MI) in cervical cancer patients with a large, nationwide cohort.

**Methods:**

The study analyzed data from the 1996 to 2010 National Health Insurance Research Database provided by the National Health Research Institutes in Taiwan. The assessed number of patients with cervical cancer with radiotherapy only, surgery with bilateral oophorectomy only, and with appendectomy were 308, 323 and 229, respectively. The Kaplan-Meier method and the Cox proportional hazards model were used to assess the risk of myocardial infarction.

**Results:**

The adjusted hazard ratio for cervical cancer in patients with MI was 1.97 (95% CI, 0.97 - 3.91; P = 0.05) for the group that received RT alone, and 2.13 (95% CI, 1.11 - 3.75; P = 0.01) for the surgery group when compared with controls. The more risk comorbidities they have, the higher the risk of myocardial infarction would be for the patients.

**Conclusion:**

The incidence of MI was significantly higher among cervical cancer patients with RT alone or surgery with bilateral oophorectomy alone than among general populations. RT might be as a factor to increase risk as bilateral oophorectomy. Whether RT itself triggers menopause or impairs the ovarian hormone production that increases the risk of MI needs to be further investigated.

## Introduction

As treatment improves, the proportion of long-term cervical cancer survivors continues to increase [1], along with concern about late-treatment-related morbidity. The relatively limited data available on late toxicity affecting cervical cancer patients treated with radiotherapy (RT) has mainly focused on gastrointestinal and urogenital toxicity [2]. Nevertheless, there is some limited information about treatment-related cardiovascular risks for these patients. For example, Maduro and colleagues recently noted that cervical cancer patients treated with RT or chemoradiation (CCRT) were at increased risk of myocardial infarction (MI) [3].

The hypothetical mechanism for the increased risk of nonfatal MI or ischemic heart disease after bilateral oophorectomy involves surgery-induced menopause [4, 5]. RT can also directly cause ovarian dysfunction, leading to impaired ovarian hormone production, followed by amenorrhea and early menopause [6, 7].

Although previous reports hinted about the possible risk of MI for cervical cancer patients after receiving RT or CCRT, the long-term effects of RT for cervical cancer patients affected by MI are still not completely understood. Thus, we designed a large-scale, nationwide, controlled cohort study to investigate whether RT increased the risk of developing MI among a subgroup of cervical cancer patients who were treated with RT alone and a subgroup of patients undergoing bilateral oophorectomy only. A group of appendectomy patients were used as controls.

## Methods

### Ethics statement

The procedures we followed were in accordance with the ethical standards of the Committee on Human Experimentation of our institution and the Helsinki Declaration. This study was approved by the Institutional Review Board at Buddhist Dalin Tzu Chi General Hospital (B10001017). The claims files include information on ambulatory care, inpatient care, gender, date of birth, and ICD codes. Since our study used anonymous secondary data, the review board waived the requirement for written informed consent from the patients involved.

### Study population

This study analyzed 1996 - 2010 data from the National Health Insurance Research Database (NHIRD), provided by the National Research Institutes in Taiwan. The NHIRD contains the medical benefit claims for 97% of the population from a registry of board-certified physicians and contracted medical facilities. Patients diagnosed with cervical cancer (ICD-9-CM code 180) between January 1996 and December 2003 were enrolled in the study. We excluded patients who had been diagnosed with cervical cancer before January 2003. We also excluded cases from the cervical cancer registry when patients had catastrophic illness and had received treatments other than RT or surgery alone. Additionally, we excluded patients who had records of MI before RT after being diagnosed with cervical cancer, and those without an identification number or date of birth. A diagnosis of cervical cancer was based on the presence of a pelvic mass noted on imaging (computed tomography, magnetic resonance imaging, and ultrasonography), and on the results of the pathology report.

Patients with a diagnosis of appendectomy (ICD OP code 47) between January 1996 and December 2003 were enrolled as a control group; these patients had demographic and clinical characteristics similar to those of the general population. We excluded patients diagnosed before January 2004, or who had a data error upon death, or a history of an MI before the diagnosis of cervical cancer; males and patients younger than 20 years of age were also excluded. Patients who were diagnosed with any type of cancer before the index date were excluded from the study.

Diagnoses of MIs were established on the basis of the patient’s medical history, clinical features at presentation, and findings from electrocardiograms and from cardiosonography, and from laboratory data. All patients and controls were followed until they developed chronic MIs (CD-9-CM code 414, 4140, 41400, 41401, 41402, 41403, 41404, 41405, 4148, 4149) until December 31, 2010. Subjects who had an MI and were diagnosed before the index date were excluded from the data analysis.

The independent variables were: age, comorbidities, geographic region, urbanization level, and socioeconomic status. Comorbidities were included hypertension (ICD-9-CM code 401-405), diabetes (ICD-9-CM code 250), and hyperlipidemia (ICD-9-CM code 272-272.4). Four geographic regions (Northern, Central, Southern, and Eastern Taiwan) and three urbanization levels (urban, suburban, and rural) were represented [8]. This study also used enrollee category (EC) as a proxy measure for the patients’ socioeconomic status (SES) because higher SES was associated with a decline in coronary heart disease (CHD) [9]. All patients were classified into 4 subgroups, from highest to lowest SES: EC1, EC2, EC3, and EC4, respectively. These variables were associated with vascular disease. In addition, four risk factors (age older than 51 years, hypertension, diabetes, and hyperlipidemia) were used to stratify the cervical cancer after treatment cohort into 3 groups: a low-risk group (no risk factor), an intermediate-risk group (1 risk factor), and a high-risk group (≥ 2 risk factors).

### Statistical analysis

The statistical software packages SAS (version 9.2; SAS Institute, Inc., Cary, NC, USA) and SPSS (version 17; SPSS Inc., Chicago, IL, USA) were used for data analysis. Between-cohort differences in frequencies of variables were evaluated using the chi-square test. Cox regression model analysis was used to calculate the effects of MI events on the study population after adjusting for confounders. The confounding factors included age, comorbidities, geographic region, urbanization level, and socioeconomic status. The vascular event-free survival was calculated using the Kaplan-Meier method. Statistical significance was established as P < 0.05.

## Results

**Figure 1:**
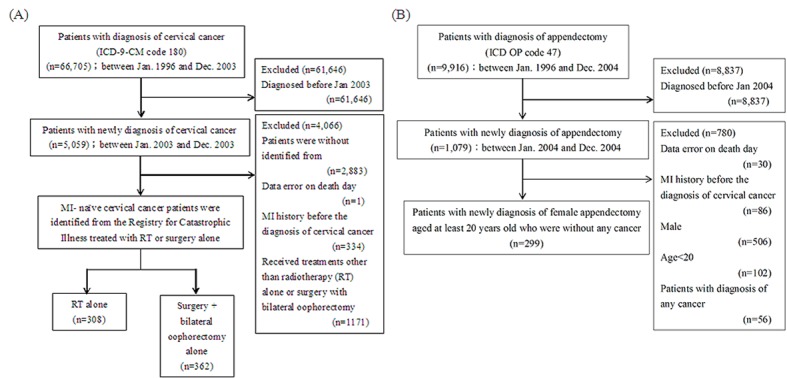
The flowcharts of (A) cervical cancer patients after treatment and (B) appendectomy patients.

The NHIRD Database listed 66,705 patients with diagnoses of cervical cancer (ICD-9-CM code 180) between January 1996 and December 2003. The number of patients who had been diagnosed as having cervical cancer records before January 2003, and thus were excluded from the study, was 61,646. Additionally, the excluded numbers of patients who had been listed in the cervical cancer registry for catastrophic illness and received treatment other than RT or surgery alone were 2,883 and 1,171, respectively. Furthermore, the patients who had records of an MI before RT after diagnosis of cervical cancer (n = 334), and those without an identification number or date of birth (n = 1) were also excluded (Fig. 1A).

There were 9,916 patients with a diagnosis of appendectomy between January 1996 and December 2003. The following patients were excluded from the study: those diagnosed before January 2004 (n = 8,837); those with data errors on death (n = 30); those with a history of an MI before the diagnosis of appendectomy was made (n = 86); males (n = 506); those younger than 20 years of age (n = 102); and subjects who had been diagnosed with any cancer before the index date (Fig. 1B) Thus, the data we assessed for the number of cervical cancer with radiotherapy only, surgery with bilateral oophorectomy only, and patients with appendectomy were 308, 323, and 229, respectively. The assessed number among those in the low-risk group, the intermediate-risk group, and the high-risk group were 285, 235, and 473, respectively. Characteristics and comorbidities of all subjects are shown in Table 1.

**Table 1:** Demographic Characteristics and Comorbidities of Cervical Cancer and Appendectomy Patients

Variable	Appendectomy (n = 299)	RT alone (n = 308)	Surgery + bilateral oopherectomy alone (n = 362)	P-value

	no. (%)	no. (%)	no. (%)	
Age (yr)				< 0.001
≤ 44	216 (72.2)	40 (13.0)	53 (14.6)	
45 - 54	50 (16.7)	63 (20.5)	153 (42.3)	
55 - 64	14 (4.7)	60 (19.5)	93 (25.7)	
65 - 74	14 (4.7)	70 (22.7)	52 (14.4)	
≥75	5 (1.7)	75 (24.4)	11 (3.0)	
Gender				NA
Female	299 (100.0)	308 (100.0)	685 (100.0)	
Hypertension				< 0.001
Yes	72 (24.1)	188 (61.0)	163 (45.0)	
No	227 (75.9)	120 (39.0)	199 (55.0)	
Diabetes				< 0.001
Yes	37 (12.4)	90 (29.2)	104 (28.7)	
No	262 (87.6)	218 (70.8)	258 (71.3)	
Hyperlipidemia				< 0.001
Yes	61 (20.4)	76 (24.7)	128 (35.4)	
No	238 (79.6)	232 (75.3)	234 (64.6)	
Geographic region				0.02
Northern	141 (47.2)	113 (36.7)	182 (50.3)	
Central	69 (23.1)	89 (28.9)	75 (20.7)	
Southern	78 (26.1)	95 (30.8)	97 (26.8)	
Eastern	11 (3.7)	11 (3.6)	8 (2.2)	
Urbanization level				0.13
Urban	88 (29.4)	68 (22.1)	108 (29.8)	
Suburban	139 (46.5)	150 (48.7)	157 (43.4)	
Rural	72 (24.1)	90 (29.2)	97 (26.8)	
EC				< 0.001
EC 1, 2	166 (55.5)	99 (32.1)	155(42.8)	
EC 3	87 (29.1)	144 (46.8)	163 (45.0)	
EC 4	46 (15.4)	65 (21.1)	44 (12.2)	

EC: enrollee category; RT: radiotherapy.

**Figure 2:**
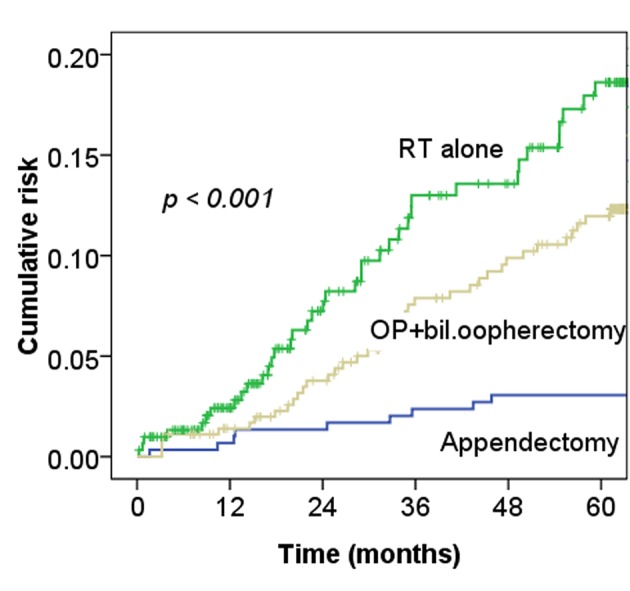
Cumulative risk of an MI for cervical cancer patients treated with RT alone or surgery (op) with bilateral oophorectomy alone and for appendectomy patients (n = 1,292).

**Figure 3:**
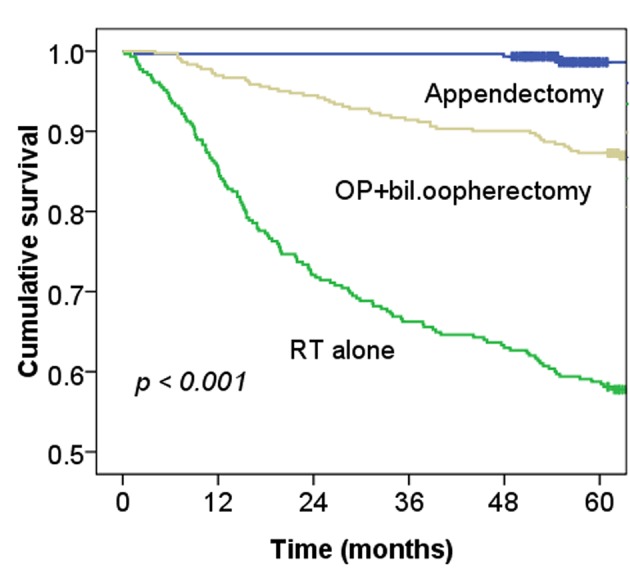
Cumulative risk of survival for cervical cancer patients treated with RT alone or surgery (op) with bilateral oophorectomy alone and for appendectomy patients (n = 1,292).

The incidence of MI was significantly higher among cervical cancer patients with RT alone than among controls (P-value < 0.001) (Fig. 2). In addition, the survival curve related to MI was worse for cervical cancer patients treated with RT alone or surgery alone than for controls (P-value < 0.001) (Fig. 3). Furthermore, the sensitivity analyses for the Cox’s model confirmed the risk of MI for cervical cancer patients treated with RT alone and remained similar after adjustment (HR 1.97, 95% CI, 0.97-3.91;P = 0.05) The risk of MI for those treated with surgery with bilateral oophorectomy was similar to the risk of MI related to treatment with RT only (Table 2).

**Table 2:** Crude and Adjusted Hazard Ratios for Myocardial Infarction (MI) During an 8-Year Follow-Up Period

	Events (%)	Unadjusted HR (95%CI)	P-value	Adjusted HR (95%CI)	P-value
Appendectomy (n = 299) (ref.)	15 (5.0)	1		1	
Radiotherapy alone (n = 308)	43 (14.0)	6.12 (3.27 - 11.45)	< 0.001	1.97 (0.97 - 3.91)	0.05
Surgery with bilateral oophorectomy alone (n = 362)	52 (14.4)	4.75 (2.58 - 8.75)	< 0.001	2.13 (1.11 - 3.75)	0.01

Adjusted for age, hypertension, diabetes, hyperlipidemia, geographic region, urbanization level, and enrollee category.

**Figure 4:**
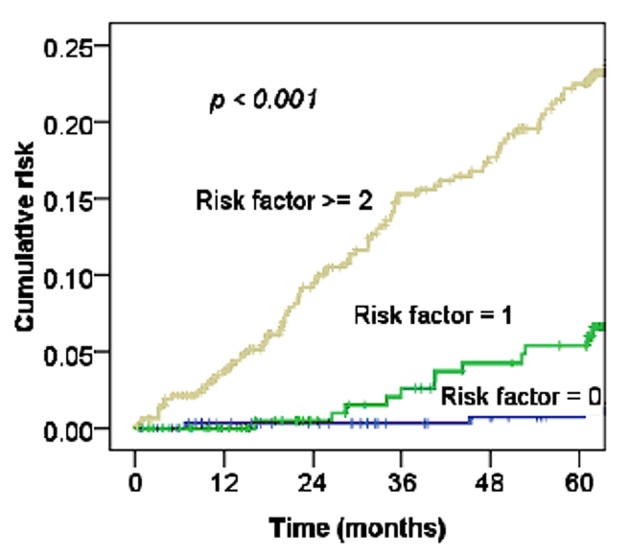
Cumulative risk of MI by stratification for factors (n = 993).

According to the risk groups of comorbidities, the more comorbidities, such as age older than 51 years, or presence of hypertension, diabetes or hyperlipidemia, the higher the rates of MI among cervical cancer patients (P < 0.001) (Fig. 4).

## Discussion

Our data suggest that, after adjusting for potentially confounding factors, the risk of MI for women with cervical cancer who were treated with RT alone was almost double that of healthy women in the general population. The data hint that the risk of an MI is similar among cervical cancer patients treated with RT as among those treated with bilateral oophorectomy. To the best of our knowledge, this study is the first large-scale, nationwide cohort trial to assess the risk of MI for subgroups of cervical cancer patients treated with RT alone.

Table 2 shows that the cervical cancer patients who received surgery with bilateral oophorectomy had an increased risk of an MI compared to normal women (HR 2.13 95% CI, 1.11 - 3.75; P = 0.01) (Fig. 2), and the worst survival of all three study groups (Fig. 3). Results of a previous study suggested that ovarian hormones have a cardioprotective effect [10]. The mechanism of surgery-induced surgical menopause is likely to be associated with the rapid rise of atherogenic lipoproteins in a reduced estrogenic state, resulting in elevated subclinical atherosclerosis [11-13]. This can be observed by increased carotid intima-media thickness after menopause, and presents as an MI after menopause [14, 15]. Data from the Framingham study showed that women who underwent oophorectomy had a 30.8% greater risk of MI than did premenopausal women [16]. Furthermore, women who undergo early bilateral oophorectomy have a risk for MI approximately 7.2 times that of premenopausal women [4]. Recently, a Danish study also showed a several-fold higher risk of ischemic heart disease associated with early removal of the ovaries [5]. Our data also confirm that bilateral oophorectomy increases the risk of MI among cervical cancer patients.

For cervical cancer patients treated with RT or CCRT, Maduro et al found an increased risk of MI compared with the general population, and 4.3% of patients died from cardiovascular events [3]. Our data also support this observation (Fig. 2, 3). The risk of CHD in women depends on the interaction of a number of factors, including increasing age, rising serum cholesterol levels, high blood pressure, SES, and diabetes mellitus [17]. Table 1 shows the relationships of MI and these factors with statistically significant differences. Moreover, as Figure 4 shows, the more risk factors cervical cancer patients have, the higher their risk of MI. Adjusted with these factors, the Cox’s model also confirmed the risk of MI related by RT for cervical cancer patients (HR 1.97, 95% CI, 0.97 - 3.91; P = 0.05).

Ionizing radiation can cause direct damage to ovarian tissue and decreases ovarian follicular reserve, leading to impaired ovarian hormone production, early menopause and amenorrhea [6, 7, 18]. In addition, the RT fields and doses also contribute to the ovarian damage. Chiarelli et al found that the women were more affected by radiation below the diaphragm, and had an increased risk of menopause or infertility [19] Stillman and colleagues found a 68% ovarian failure rate among women who had both ovaries within the abdominal RT fields. In addition, the researchers found that the odds for ovarian failure when both ovaries were irradiated were 19.7 times higher than for other irradiated patients [20].

Chemaitilly et al found that 54% of survivors of childhood cancer who received at least 10 Gy to the ovaries during abdominal or pelvic irradiation would eventually have acute ovarian failure [21]. Additionally, Chiarelli et al noted that women treated with radiation does greater than 35 Gy had a 32% greater fertility deficit than did groups who received lower doses (P = 0.017) [19]. However, it could be argued that the RT fields and doses were not recorded in a current database. According to Wallace et al’s report, the radiosensitivity of the human oocyte is less than 2 Gy [22]. Furthermore, pelvic or pelvic plus para-aortic irradiation only applied to cervical cancer patients is generally 40 to 50 Gy [23]. Thus, the possibility of impaired ovarian and hormone production caused by RT increasing the risk of MI for cervical cancer patients can’t be ignored.

Our study had several limitations. First, the diagnosis of cervical cancer, and any other comorbid condition, was completely dependent on ICD codes. The Bureau of National Health Insurance in Taiwan randomly reviewed records and interviewed patients in order to verify the accuracy of the diagnosis. Second, some of the lifestyle characteristics, such as diet, smoking, alcohol intake, body mass index and physical activity, are correlated with CHD but this information was not in our database [17]. Indeed, this fact might tend to lead to an underestimate of the true effect. However, two diet-dependent risk factors, hyperlipidemia and hypertension, may be viewed not only as etiologically significant traits, but also as markers of other lifestyle characteristics [24]. In addition, the health benefits of higher SES are mediated through lifestyle variables [17]. These factors are all included as confounders to explain the increased risk of MI for cervical cancer patients treated with RT alone. Furthermore, our data also didn’t record the factors related to CHD, such as menopausal status or levels of ovarian hormone and hormone therapy [25]. However, we factored in age, using the age of 51 years to decrease the possibility of underestimating the effects of menopause, which on average occurs at approximately 51 years [26]. Fourth, cancer stages were not included, because this information was not available from the database. Instead of cancer-specific survival rates, overall survival rate was used because it was not possible to determine cause-specific survival rates based on the registry data.

Newer radiation techniques, including intensity-modulated RT, helical tomotherapy and proton RT, may mitigate these radiation-related treatment effects by avoiding direct irradiation to the ovaries; however, these require further investigation [27-29]. And, the role of hormone therapy for decreasing risk of CHD in early natural or surgical menopause still remains controversial [5, 25]. In our study, the possible role of hormone therapy among cervical cancer patients with MI related to RT or oophorectomy can’t be answered. However, the current analysis does shed new light on prior data and confirms the risk of MI related to for RT or bilateral oophorectomy for cervical cancer patients. It may be useful for prospective clinical trial planning.

In summary, clinicians should be aware that an MI in a cervical cancer patient could be associated with RT or surgery plus bilateral oophorectomy alone. The more comorbidities a cervical cancer patient has, the greater her risk for having an MI. Whether RT itself accelerates menopause or impairs the ovarian hormone production that causes the increasing risk of MI needs to be further investigated.
